# A personalized and dynamic risk estimation model: The new paradigm in Barrett’s esophagus surveillance

**DOI:** 10.1371/journal.pone.0267503

**Published:** 2022-04-27

**Authors:** Carlijn A. M. Roumans, Manon C. W. Spaander, Iris Lansdorp-Vogelaar, Katharina Biermann, Marco J. Bruno, Ewout W. Steyerberg, Dimitris Rizopoulos, on behalf of the ProBar study group

**Affiliations:** 1 Department of Gastroenterology & Hepatology, Erasmus MC University Medical Center, Rotterdam, The Netherlands; 2 Department of Public Health, Erasmus MC University Medical Center, Rotterdam, The Netherlands; 3 Department of Pathology, Erasmus MC University Medical Center, Rotterdam, The Netherlands; 4 Department of Biomedical Data Sciences, Leiden University Medical Center, Leiden, The Netherlands; 5 Department of Biostatistics, Erasmus MC University Medical Center, Rotterdam, The Netherlands; University of Michigan, UNITED STATES

## Abstract

**Objectives:**

The current surveillance strategy in Barrett’s esophagus (BE) uses only histological findings of the last endoscopy to assess neoplastic progression risk. As predictor values vary across endoscopies, single measurements may not be an accurate reflection. Our aim was to explore the value of using longitudinal evolutions (*i*.*e*. successive measurements) of histological findings (low-grade dysplasia (LGD)) and immunohistochemical biomarkers (p53 and SOX2) by investigating the association with Barrett’s progression.

**Methods:**

In this proof-of-principle study of a longitudinal dynamic risk estimation model with a multicenter cohort design, 631 BE patients from 15 Dutch hospitals who were under surveillance were included. Longitudinal dynamic values of LGD, p53, and SOX2 were included in a multivariate joint model to estimate the risk of high-grade dysplasia (HGD)/esophageal adenocarcinoma (EAC).

**Results:**

Longitudinal evolutions of aberrant expression of p53 (HR 1.26, p<0.01) and SOX2 (HR 1.43, p<0.01) were associated with an increased HGD/EAC risk. We also found weak evidence of an association with the longitudinal evolution of the presence of LGD (HR 1.02, p = 0.12). The performance of the model was good (AUC 0.80–0.88). Using this model, for each future BE patient the probability of aberrant expression of biomarkers based on multiple longitudinal observations can be estimated. This probability is translated in progression risk, expressed as HR.

**Conclusions:**

This study provides solid ground to further explore a paradigm shift from currently recommended fixed intervals towards personalized surveillance, in which tailored risk estimations and corresponding surveillance intervals can be updated at every FU endoscopy for individual BE patients.

## Introduction

Barrett’s esophagus (BE) is a premalignant condition of esophageal adenocarcinoma (EAC). Progression is expected to be stepwise: from no dysplasia (NDBE) to low-grade dysplasia (LGD), eventually to high-grade dysplasia (HGD) and EAC. In the natural history EAC is usually detected in a more advanced stage due to the late onset of symptoms, which causes high mortality [1]. Surveillance by upper endoscopy was introduced to detect neoplasia in an earlier (asymptomatic) and endoscopically treatable stage, which is likely to reduce EAC related morbidity and mortality [2–5].

To date, studies show conflicting results about the effectiveness of surveillance [6–9]. The main predictor to identify an increased risk of developing neoplasia is the presence of LGD in biopsy specimens, as sampled during surveillance endoscopy. If present, the surveillance interval is shortened.

However, the discriminative power between patients with low and high risk of neoplastic progression is limited: only an estimated 15% of patients in whom LGD was detected will develop HGD/EAC [10]. Consequently, the majority of patients with a supposedly increased risk are having surveillance endoscopies too frequently. Current approaches only use the most recent available assessment of histological diagnosis to estimate the neoplastic progression risk [11, 12]. As a result, this strategy does not account for the fact that the presence or absence of LGD can change dynamically over time within one patient. Previously, persistent LGD was estimated to be associated with a higher neoplastic progression risk than a single measurement of LGD. However, in those models it was used as a single dichotomous parameter [13, 14]. Therefore, these previous models may be inaccurate.

There is a need for an alternative strategy to improve risk stratification. First of all, longitudinal evolutions (successive measurements within the same patient) of the histological diagnosis over time can provide additional information in estimating neoplasia risk [15], rather than single measurements or multiple measurements defined a dichotomous parameter. This strategy allows for an updated (dynamic) personalized risk estimation at every follow-up (FU) endoscopy, thereby including the history of the individual BE patient. Personalized (and potentially longer) surveillance intervals can be initiated, as well as termination of surveillance in case of very low risk. Secondly, besides histological assessment, other biomarkers need to be implemented in risk estimations for neoplastic progression. For two immunohistochemical biomarkers strong potential has been shown: p53 [10, 16] and SOX2 [17].

The aim of this proof-of-principle study was to explore the value of using these longitudinal profiles of histological diagnosis, p53, and SOX2 by investigating the association with neoplastic progression. If present, this may cause a shift of paradigm in BE surveillance from fixed surveillance intervals based on only the most recent measurement of histological diagnosis towards personalized surveillance based on longitudinal evolutions of three biomarkers with a more precise and reliable risk estimation.

## Materials and methods

### Study design

The design of this multicenter cohort study has been described previously [17]. In summary, consecutive BE patients from 15 Dutch hospitals (three university hospitals and 12 general hospitals) were included between September 2003 and December 2004. At index endoscopy demographic information, such as age and gender, was collected. Before every FU endoscopy, patients were asked to fill out a questionnaire, concerning weight, length, gastro-esophageal reflux symptoms, use of medication, smoking, and alcohol use. During endoscopies landmarks were identified, as well as the presence of visible abnormalities, and the presence of esophagitis [18]. Surveillance intervals were according to the guideline of the American College of Gastroenterology, and biopsies were sampled randomly as stated by the Seattle protocol. The endpoint of the study was the detection of HGD/EAC.

### Study population

Inclusion criteria were histologically confirmed intestinal metaplasia in biopsies obtained from columnar lined epithelium in the esophagus, Barrett segment ≥2 cm, and absence of a history of HGD/EAC. To exclude prevalent cases of neoplasia at baseline, only BE patients with ≥6 months of FU in the study without HGD/EAC development were selected for this analysis. Therefore, results concerning median FU time and the incidence rate of (HGD/)EAC were based on total FU time minus six months per patient. Immunohistochemistry results were obtained from biopsies of each endoscopy of patients who had eventually developed HGD/EAC and from one random endoscopy of patients without HGD/EAC. Although some patients had only one measurement per biomarker, it is possible to take into account the longitudinal profile based on all patients. If there was no paraffin material available of any endoscopy, patients were excluded.

### Histology and immunohistochemistry

The histological diagnosis and the expression of p53 and SOX2 immunohistochemistry was assessed by at least two experienced investigators in the biopsy specimens as sampled during surveillance endoscopy. The working experience of pathologist 1 (KB) after graduation was 12 years, with a BE caseload of 8 up to 10 weekly; pathologist 2 was working 8 years after graduation, assessing 8 up to 10 BE cases weekly as well. The highest degree of abnormality in a biopsy set was reported (S1 Appendix).

### Ethics

The institutional review board of Erasmus MC University Medical Center Rotterdam, The Netherlands, and the board of all participating centres approved the study protocol. Written informed consent was gained from patients at inclusion.

### Statistical analysis

Neoplastic progression was defined as the development of HGD/EAC. The biomarkers investigated were all considered dichotomous and either normal or aberrant: NDBE vs LGD, normal vs aberrant staining of p53 (overexpression or loss of expression), and normal vs loss of expression of SOX2. To investigate the association between biomarkers and the development of neoplasia a multivariate joint model was used [19]. In this model, longitudinal data of the risk of aberrant measurements of a biomarker are combined with the risk of neoplastic progression.

First, the risk of showing LGD or aberrant expression of p53 or SOX2 was modelled for each of the three biomarkers separately with mixed-effects logistic regression models, based on the course of longitudinal evolutions. We assumed results of indefinite for dysplasia as missing values of histopathology. For all three longitudinal models (LGD, p53, SOX2) in the fixed part the baseline variables age and gender, and the time-varying covariates time, esophagitis, and BE length were included. Age was standardized. Esophagitis was considered dichotomous, to be either present or absent. BE length was also considered dichotomous with short-segment BE <3 cm and long-segment BE ≥3 cm. In the random-effects part for each model only random intercepts were used. Secondly, these models were combined with a time-varying Cox proportional hazards model in the framework of a multivariate joint model, to estimate the dynamic risk of neoplastic progression. Both the Cox model and the multivariate joint model were adjusted for baseline values age and gender, and time-varying dichotomous covariates BE length and esophagitis.

To estimate the predictive performance the area under the receiver operating characteristic curve (AUC) was determined for neoplastic progression risk predictions within three years at time point year one, two, three, four, five, and six. Internal validation using the Bootstrap method with 100 replications was performed to adjust for optimism.

Odds ratios (OR) >1 of the mixed-effects logistic regression models are associated with an increased risk of aberrant expression of biomarkers. Hazard ratios (HR) >1 of the multivariate joint model are associated with an increased risk of neoplastic progression. P-values <0.05 were considered statistically significant. Analysis was done with R [20], version 3.4.1, using package JMbayes [21].

### Sensitivity analysis

To compare the estimates to more conventional analysis, a static Cox proportional hazards model was estimated using the same data and variables. Further details can be found in the S1 Appendix.

## Results

### Patient characteristics

Out of 728 patients meeting the inclusion criteria, 631 (87%) were included (S1 Fig), in whom 3276 endoscopies were performed. For the 97 (13%) patients excluded there were no results available of any FU moment for LGD, p53, and SOX2 (Table 1).

**Table 1:** Baseline characteristics.

Variables		Patients included (n = 631)	Patients excluded (n = 97)	p-value
FU time (median, IQR)		6.8 years (4.9–9.8)	8.0 years (2.4–10.9)	0.95
n° of FU (median, IQR)		**4.0 (3.0–6.0)**	**4.0 (2.0–5.0)**	***<0*.*01***
Age (median, IQR)		60 years (53–69)	62 years (53–70)	0.60
Male gender (%)		463 (73%)	68 (70%)	0.58
GERD (%)		192 (30%)	30 (31%)	1.00
PPI use (%)		565 (90%)	91 (94%)	0.29
NSAID use (%)		32 (5.1%)	3 (3.1%)	0.55
Smoking (%)	current	133 (21%)	14 (14%)	
	ever	287 (45%)	43 (44%)	
	never	199 (32%)	40 (41%)	0.12
Alcohol (%)	current	**488 (77%)**	**66 (68%)**	
	ever	**58 (9.2%)**	**8 (8.2%)**	
	never	**73 (12%)**	**23 (24%)**	***<0*.*01***
BMI (median, IQR)		26.6 kg/m^2^ (24.6–29.2)	26.3 kg/m^2^ (24.0–29.7)	0.84
HGD/EAC		54 (8.6%)	3 (3.1%)	0.10
Length of BE ≥3 cm (%)		**478 (76%)**	**60 (62%)**	***<0*.*01***
Esophagitis present (%)		61 (10%)	14 (14%)	0.20

BMI = body mass index. BE = Barrett’s esophagus. FU = follow-up. GERD = gastro-esophageal reflux disease. HGD = high-grade dysplasia. NSAID = non-steroidal anti-inflammatory drug. PPI = proton pump inhibitor. EAC = esophageal adenocarcinoma.

The mean number of measurements per biomarker for patients who had eventually developed HGD/EAC was for LGD 4.1 (SD 2.2), for p53 3.8 (SD 2.1), and for SOX2 3.7 (SD 2.0). In patients who had not developed HGD/EAC in the study this mean number of measurements was for LGD 5.0 (SD 1.7), for p53 2.2 (SD 2.0), and for SOX2 2.2 (SD 2.0).

4475 person-years were observed in patients included; 54 patients developed HGD/EAC. The average incidence rate was 1.2 (95% CI 0.9–1.5) for HGD/EAC and 0.4 (95% CI 0.2–0.6) for EAC per 100 person-years. The median FU time was 6.8 years (IQR 4.9–9.8), with a median age of 60 years (IQR 53–69). Predominantly males were included (73%) and 76% of patients had a BE segment of ≥3 cm.

### Risk estimation of aberrant measurements of biomarkers

The longitudinal evolutions of histological diagnosis and immunohistochemistry were estimated based on measurements of multiple successive endoscopies within the same patient (Table 2). The risk of detection of LGD, instead of NDBE, was increased due to older age (OR 1.58, p<0.01) and male gender (OR 0.55, p<0.01 (ref. males)). The risk of aberrant expression of p53 increased in time (OR 1.17, p<0.01), with older age (OR 1.82, p<0.01), and male gender (OR 0.23, p<0.01 (ref. male)), but also with a long segment BE (OR 2.77, p<0.01). Loss of SOX2 expression was not influenced by any of these factors. Absolute measurements of the variability between normal and aberrant expression can be found in S2 and S3 Tables.

**Table 2:** Risk of aberrant expression of biomarker. Longitudinal models: ORs and 95% CI of the risk of having LGD, aberrant expression of p53, or SOX2 in time, adjusted for age, gender (at baseline), length of BE, and esophagitis (time-varying covariates). OR >1 indicate an increased probability of LGD or aberrant expression of p53 or SOX2 if the mentioned characteristic is present.

	LGD	p53	SOX2
	OR (95% CI)	OR (95% CI)	OR (95% CI)
**Time**	1.00 (0.96; 1.04)	**1.17 (1.06; 1.29)**	1.09 (1.00; 1.19)
**Age**	**1.58 (1.31; 1.91)**	**1.82 (1.21; 2.72)**	1.28 (0.98; 1.67)
**Gender (female)**	**0.55 (0.36; 0.82)**	**0.23 (0.09; 0.58)**	0.84 (0.47; 1.49)
**Length of BE (≥3 cm)**	1.19 (0.84; 1.67)	**2.77 (1.38; 5.56)**	1.13 (0.67; 1.90)
**Esophagitis (present)**	1.08 (0.63; 1.87)	0.45 (0.16; 1.28)	0.76 (0.32; 1.79)

BE = Barrett’s esophagus. LGD = low-grade dysplasia. OR = odds ratio.

### Risk estimation of neoplastic progression

The risk of neoplastic progression was estimated based on the longitudinal evolution of LGD, p53, and SOX2 (Table 3). For the results of the biomarkers of the multivariate joint model the *value* (representing the current risk of neoplastic progression, based on all previous measurements) and the *accumulated effect* of this biomarker (representing the overall risk of neoplastic progression, based on the history of the measurements of biomarkers) are reported. In clinical practice, this difference is irrelevant and the interpretation of the results is comparable; in both risk estimations, all biomarker measurements of each patient are included. However, the mathematical approach is different. Consequently, both value and the accumulated effect can be used in estimating the neoplastic progression risk.

**Table 3:** Risk of neoplastic progression. For every biomarker normal expression is the reference category. HR >1 is associated with an increased risk; HRs of joint model represent the HGD/EAC risk if the risk of aberrant expression in the longitudinal course of a biomarker changes with 10%, HRs of the Cox model represent the HGD/EAC risk if aberrant expression is present at baseline.

		Joint model	Cox model
		HR (95% CI)	HR (95% CI)
Age		1.00 (0.98; 1.05)	1.19 (0.86; 1.63)
Gender (female)		1.00 (0.90; 1.02)	0.77 (0.37; 1.58)
Length of BE (≥3 cm)		1.02 (0.94; 1.03)	1.04 (0.54; 2.03)
Esophagitis (present)		1.03 (0.75; 1.02)	**3.38 (1.67; 6.85)**
LGD	Value	1.02 (0.47; 1.50)	**3.57 (2.01; 6.34)**
Accumulated effect		1.02 (1.00; 1.06)	n.a.
P53 (aberrant expression)	Value	**1.26 (1.13; 1.80)**	**6.63 (3.55; 12.4)**
Accumulated effect		1.00 (1.00; 1.00)	n.a.
SOX2 (aberrant expression)	Value	**1.43 (1.26; 3.23)**	**2.20 (1.12; 4.34)**
Accumulated effect		1.02 (1.00; 1.05)	n.a.

BE = Barrett’s esophagus. HGD = high-grade dysplasia. HR = hazard ratio. LGD = low-grade dysplasia. EAC = esophageal adenocarcinoma.

An increased risk of developing HGD/EAC during surveillance was associated with aberrant expression of p53 (HR value 1.26, p<0.01) or SOX2 (HR value 1.43, p<0.01). The results of LGD (HR value 1.02, p = 0.78 & HR accumulated effect 1.02, p = 0.12) were not statistically significant. Consequently, there may be an association with an increased risk of neoplastic progression and LGD, but the association with aberrant expression of p53 or SOX2 is considerably larger. This indicates that multiple successive aberrant measurements of p53 or SOX2 increase the risk of developing HGD/EAC.

**Fig 1:**
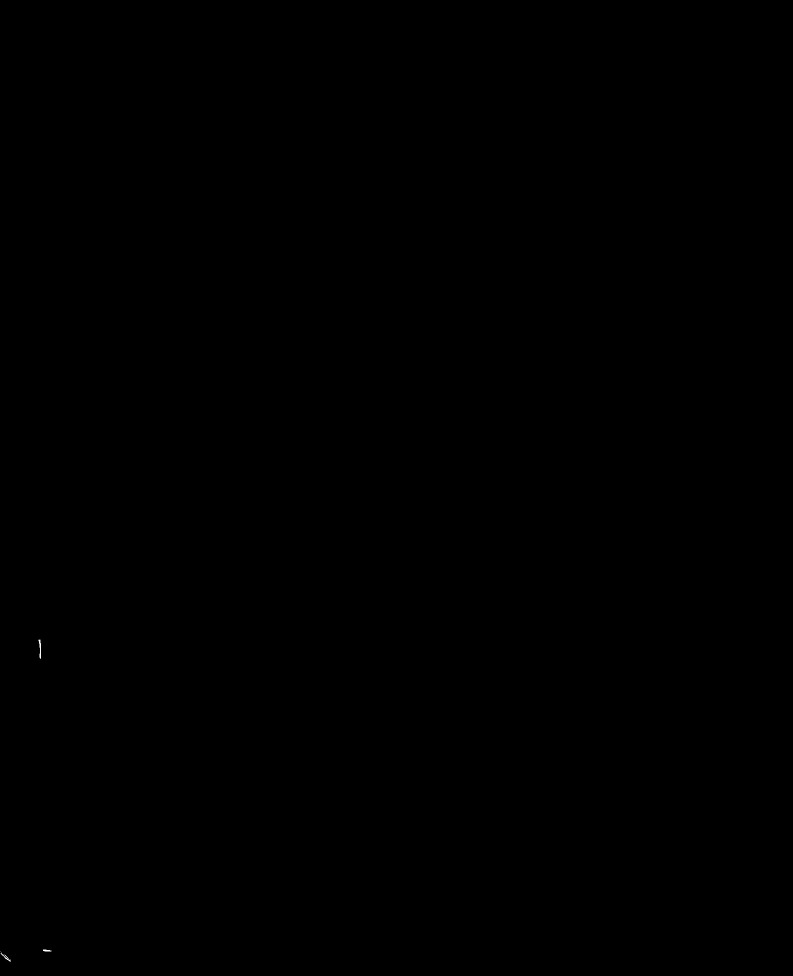
Personalized HGD/EAC risk estimations for a BE surveillance patient, based on age, gender, esophagitis, and BE length. Additionally, at every follow-up markers LGD, p53, and SOX2 are tested, and HGD/EAC risk estimations within three years are updated, based on all measurements of that patient. Based on these dynamic risk estimations the interval can be either shortened, or endoscopic eradication therapy can be applied. BE = Barrett’s esophagus. HGD = high-grade dysplasia. LGD = low-grade dysplasia. EAC = esophageal adenocarcinoma.

**Fig 2:**
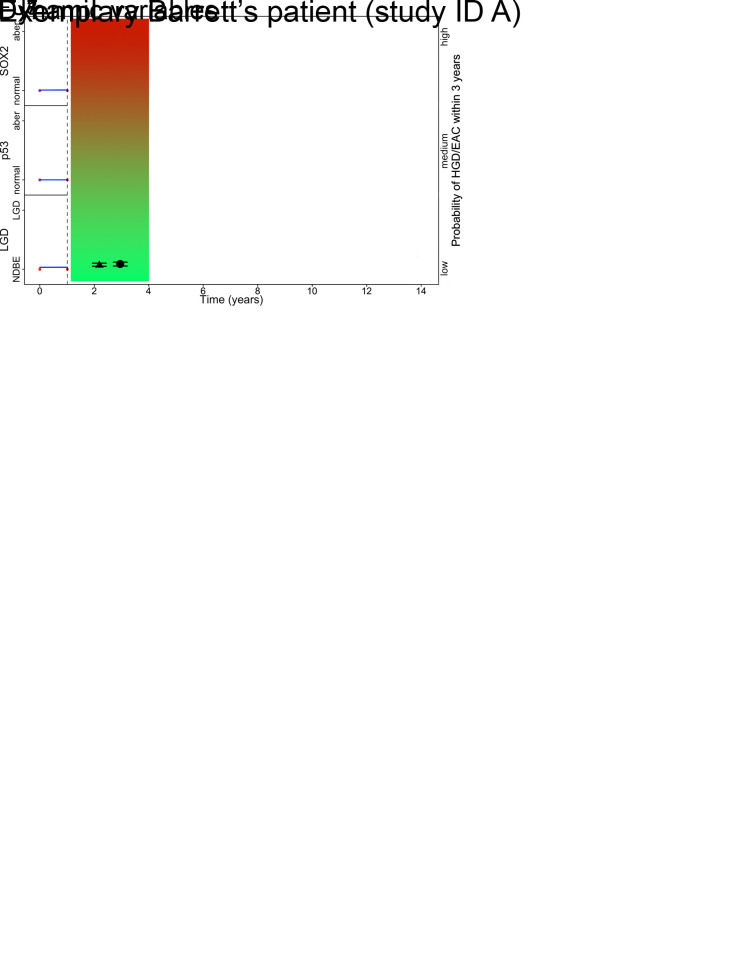
Neoplastic progression risk estimation of an exemplary BE patient (black bars, 95% CI) within three years at different FU moments (1, 3, 7), based on: 1) baseline variables (gender, age, esophagitis, BE length); 2) dynamic variables (red points represent measurements of three biomarkers, blue lines represent the probability of aberrant measurements in time). The difference between HGD/EAC risk estimations based on the dynamic and static model are displayed. In the static model the risk is only based on the last measurement. However, for the dynamic model the risk can be adjusted to all measurements; if there are more normal biomarker measurements, the probability of developing HGD/EAC settles (*e*.*g*. in FU3). BE = Barrett’s esophagus. FU = follow-up. HGD = high-grade dysplasia. EAC = esophageal adenocarcinoma.

The HRs in Table 3 represent the probability of HGD/EAC in a patient with only normal measurements of that specific biomarker, compared to a patient with only aberrant measurements. In practice, these measurements will differ across endoscopies; one individual patient will not have only normal or only aberrant measurements. Therefore, first the probability of having LGD or aberrant expression of p53 or SOX2 has to be estimated based on the longitudinal evolution (*i*.*e*. successive measurements) (Table 2). This will provide an individual proportion of the total estimated HGD/EAC risk (Table 3). For example, the risk of aberrant expression of p53 is ~20% in case of five measurements: four normal, one aberrant. The HGD/EAC risk of that individual patient at that time point based on only p53 is 0.2*1.26 = HR 0.25. If the model will be used in an online application, it will include all demographic and clinical variables of that individual patient, as well as the longitudinal evolutions of histological diagnosis, p53, and SOX2 to estimate the neoplastic progression risk. The risk estimations can be updated at every surveillance endoscopy, based on new additional measurements of histological diagnosis and immunohistochemistry. This results in dynamic risk estimations for each patient, according to its individual patient characteristics (Figs 1 and 2). These risks for the development of HGD/EAC gradually evolve from low risk to high risk. Each risk will have its consequence in surveillance, for example for low risk the interval can be chosen to remain long (*e*.*g*. three years), for medium risk the interval could be shortened (*e*.*g*. towards one year), and for high risk patients endoscopic eradication therapy (EET) may be applied.

### Predictive performance

Optimism-adjusted estimates of the AUC were between 0.80 and 0.88 at different time points, indicating good performance of the model. Since the estimates before adjustment for optimism did not deviate much from those afterwards, there were no signs of overfitting (Table 4).

**Table 4:** Validation personalized risk estimation model: 1) area under the curve (AUC) of the dynamic model (= joint model), 2) AUC of the dynamic model, adjusted for optimism, 3) AUC of the static model (= Cox model). All estimates were measured at time points year 1, 2, 3, 4, 5, and 6.

Year	AUC dynamic model	AUC dynamic model adjusted for optimism	AUC static model
1	0.89	0.88	0.78
2	0.84	0.80	0.75
3	0.88	0.84	0.78
4	0.87	0.84	0.78
5	0.88	0.87	0.76
6	0.87	0.86	0.72

AUC = area under the receiver operating characteristic curve.

### Sensitivity analysis

In the static model the presence of LGD, aberrant expression of p53 and SOX2 were associated with an increased risk of neoplastic progression (HR LGD 3.57, p<0.01, HR p53 6.63, p<0.01; HR SOX2 2.20, p = 0.04) and the presence of esophagitis (HR 3.38, p<0.01). The estimates of the AUC at different time points were all lower than those of the main analysis (0.72–0.78, Table 4). If in the same static model only baseline LGD was included and not p53 and SOX2, there was a statistically significant association with an increased risk of neoplastic progression (HR 3.40, p<0.01). Further details can be found in the S1 Appendix and S1 Table.

## Discussion

In this first ‘proof-of-principle’ study the value of using longitudinal profiles of biomarkers to estimate the risk of Barrett’s progression was shown. Successive measurements of aberrant expression of p53 and SOX2 were associated with an increased risk; the longitudinal evolution of the presence of LGD may also be associated. Demographic and clinical variables as age, gender, and BE length are involved as well. The predictive performance of this dynamic model was shown to be higher than the static model. Consequently, this study provides solid ground to further explore a potential shift of paradigm from the current guideline-recommended intervals, which are fixed, towards personalized surveillance. These tailored risk estimations, based on an individuals’ longitudinal measurements, can be used to predict the neoplastic progression risk of each BE patient to schedule their next endoscopy. This interval can vary per patient, but also per endoscopy within the same patient if new biomarker measurements are included and the neoplastic progression risk is updated.

There are two important benefits of this model. First of all, all successive measurements of every single patient were used. Consequently, biomarkers could be used in the model as if they were measured continuously. Since it is known that measurements of biomarkers can vary within one patient across endoscopies, this reduces the influence of an incorrect histological diagnosis. This is most likely to be caused by misinterpretation, as concurrent inflammatory changes are difficult to distinguish from LGD due to reactive atypia. Other contributing factors are the influence of artifacts as well as sampling error. Although the latter is unlikely to play an important role if endoscopists adhere to the biopsy protocol, e.g. the Seattle protocol, as recommended by guidelines. Another option is that instead of having false-negative or false-positive results for the presence of low-grade dysplasia due to the previously mentioned reasons, actual regression and progression of the grade of dysplasia is a possibility. Although heavily discussed in the literature, the general opinion is that regression is unlikely to play a role [22, 23]. Secondly, since this model provides more information about longitudinal evolutions of biomarkers; it allows the inclusion of the history of the patient and its cumulative neoplastic progression risk. Due to these benefits, the intended model is likely to provide a better representation of the relevant findings over time and we expect it to be more accurate: in our study the AUC of the dynamic model was better than the AUC of the static model.

Currently, in BE surveillance guidelines the recommended interval is shortened if LGD is determined in biopsy specimens [2]. These recommendations are in line with a recent meta-analysis, which showed that the HGD/EAC risk was increased more than four times in case of LGD detection [24]. However, only a minority of patients with LGD will eventually develop HGD/EAC [10]. The latter patients, who are false-positive for high neoplastic progression risk, have to undergo many unnecessary endoscopies, or even EET. A static model based on our data with only baseline LGD included, also showed an increased HGD/EAC risk of almost four times. However, in our risk estimation model, including longitudinal evolutions of biomarkers, with the same data, having LGD was not as strongly associated with an increased neoplastic progression risk. These findings support the impact of misclassification of the histological diagnosis, leading to an erroneously estimated increased risk. If LGD is detected multiple times, the likelihood of a true-positive measurement for LGD and its predictive value for neoplasia is likely to be higher [25].

Nowadays, patients with HGD or early EAC are treated with EET. However, there is a trend towards EET for patients with LGD [23]. If all LGD patients would be having EET, the patient burden of treatment would be unnecessary for approximately 70% of the patients [26]. Targeted treatment to only high-risk patients based on personalized surveillance seems appropriate, to which the model in this study may contribute. Besides, this model may have a beneficial effect on the number of endoscopies needed per patient. Eventually the cost-effectiveness of BE surveillance could improve with a reduced healthcare burden.

Apart from the fact that in this model we have used longitudinal evolutions, immunohistochemical biomarkers that have already shown promising results in the literature have been included. P53 is expected to play an early role in the progression of BE towards neoplasia, as mutations are already noted in biopsies showing no dysplasia. Its role in neoplastic progression is assumed to be due to inactivation of tumor suppression, but probably mostly due to genome doubling ending up in oncogenic amplifications [27]. In a meta-analysis containing 1340 BE patients the odds of neoplastic progression were estimated to be three times higher if p53 was expressed aberrantly, compared to the wild-type [16]. Although SOX2 is not as extensively researched as p53, a case-control study by our group using the same data showed a relative risk of almost five in case of loss of expression of SOX2 compared to normal expression [17]. SOX2 is a transcription factor with an important role for a stem cell to remain pluripotent and consequently in esophageal differentiation; mutations in SOX2 indicate dedifferentiation with a higher risk of malignant transformations as a consequence [28]. These results are in line with our findings, as the longitudinal trajectories of aberrant expression of both p53 and SOX2 were associated with increased risk of the development of neoplasia. There are also practical benefits to the additional use of these biomarkers. Since they can be analysed from formalin fixed biopsy specimens, no alternative procedures are necessary to process the material, besides staining. Therefore, the additional costs and extra efforts are likely to be small and not time consuming. Furthermore, the interobserver rate to define p53 expression was estimated to be rather good, with a κ-value of 0.71, compared to 0.24 in the same study for LGD [29]. Also, p53 has been shown to improve interpretation of histological diagnosis by pathologists, if it were to be used as an ancillary study [30]. In the future, it may even be possible to determine the expression of p53 automatically, as is currently evaluated in other fields of research [31].

There are several strengths and limitations to this study. Because of the use of longitudinal evolutions of biomarkers, these measurements seem to be measured continuously. Consequently, the influence of missing data is lower than if only single measurements were used. Besides, for that same reason the influence of patients dropping out for a reason related to a higher risk of neoplastic progression is reduced. For example, if a patient drops out due to increased age, this patient may have developed neoplasia, but it was not observed. Since the longitudinal evolutions of biomarkers are modelled, the longitudinal course can be extrapolated and the event can be ‘observed’ anyway. There is, however, a difference in the mean number of measurements per biomarker between those patients who eventually show neoplastic progression and those patients who do not. This may be a potential cause of bias. Due to lack of availability of the tissue of the biopsy specimens it was not possible to collect more data. We have included all available information, since we believe the increment of the power of the model is higher if we do use all data available than the potential risk of bias if we would not have used them. Besides, the model itself adjusts for this potential risk. In cases of patients with more biomarker measurements, the model recognizes an increased risk, which is incorporated in the estimations of the hazard of developing neoplastic progression [32]. Data were collected in a multicenter design, in both university and general hospitals. Therefore, this cohort is likely to be representative to clinical practice. All biopsy specimens were reviewed by two pathologists experienced in gastroenterology, which is likely to reduce the influence of misclassification. However, since the first data were collected in 2003 and since the expertise of assessing histological diagnosis has improved along the years, some results may be outdated. Also, due to the low incidence of neoplasia, in our cohort 54 events were observed. Although this is a unique cohort of BE patients including many endoscopies and even more biopsies, for statistical modelling this still represents a limited number of events. In the model, adjustments were made to prevent it from overfitting. Consequently, more information (confounders, variable risks in time) was left out, that could have improved the quality of the model.

In conclusion, longitudinal evolutions of immunohistochemical biomarkers and, to a lesser extent, histological diagnosis are of great value in risk stratification to predict Barrett’s progression. The next phase will be to make a prediction model based on these associations to be able to provide tailored intervals for each individual BE patient in the framework of a personalized surveillance program. A ready-to-use tool in an online web application in which all measurements from a patient are entered, will provide an instant (updated) personalized risk prediction with corresponding surveillance interval.

## Supporting information

S1 Appendix (DOCX)

S1 Fig Flow chart of patients included. EAC = esophageal adenocarcinoma. LGD = low-grade dysplasia. HGD = high grade dysplasia.
(DOCX)

S1 Table Risk of neoplastic progression. For every biomarker normal expression is the reference category. HR >1 is associated with an increased risk; HRs of joint model represent the HGD/EAC risk if the risk of aberrant expression in the longitudinal course of a biomarker changes with 10%, HRs of the Cox model represent the HGD/EAC risk if aberrant expression is present at baseline. BE = Barrett’s esophagus. HGD = high-grade dysplasia. HR = hazard ratio. LGD = low-grade dysplasia. EAC = esophageal adenocarcinoma.
(DOCX)

S2 Table Absolute measurements of the variability between normal and aberrant expression, not related to individual patients. (DOCX)

S3 Table Absolute measurements of the variability between normal and aberrant expression for SOX2, not related to individual patients. (DOCX)

## Abbreviations

AUC: Area under the receiver operating characteristic curve
BE: Barrett’s esophagus
EET: endoscopic eradication therapy
EAC: esophageal adenocarcinoma
FU: follow-up
HR: hazard ratio
HGD: high-grade dysplasia
LGD: low-grade dysplasia
NDBE: no dysplasia
OR: odds ratio
