# Supplementary material for: A personalized and dynamic risk estimation model: The new paradigm in Barrett’s esophagus surveillance
Source: PLoS One. 2022 Apr 27;17(4):e0267503. doi: 10.1371/journal.pone.0267503 (PMC9045660; doi:10.1371/journal.pone.0267503)
Supplement: S1 Appendix — (DOCX) [file pone.0267503.s001.docx]

**Appendix**

Histology and immunohistochemistry

The histological diagnosis and the expression of p53 and SOX2 immunohistochemistry was assessed in the biopsy specimens as sampled during surveillance endoscopy. The highest degree of abnormality in a biopsy set was reported.

First of all, to determine the presence of intestinal metaplasia and the histological diagnosis, haematoxylin eosin slides were examined consecutively by a local and an expert pathologist. If inconclusive, a second expert pathologist reviewed the slides. A panel of pathologists reviewed the slides afterwards in case there was still disagreement, to reach a final consensus agreement about the histological diagnosis.

Secondly, the immunohistochemical slides were examined. For p53, if at least one gland showed overexpression or complete loss of expression, it was considered aberrant. Otherwise, the expression was considered normal [10]. For SOX2, loss of expression in a cluster of glands was considered aberrant, except for the glands with many goblet cells. Strong and weak nuclear SOX2 positivity was considered normal expression [15]. The immunohistochemical slides were scored in tandem with the haematoxylin eosin slides by two experienced investigators, if present, in areas with dysplasia. They were blinded for the presence of neoplastic progression in the long-term. If there was any disagreement, both investigators reviewed the slides simultaneously to reach a final consensus about the diagnosis.

Sensitivity analysis

Besides the main analysis, two sensitivity analyses were performed. First of all, the main analysis was repeated by including only the mixed effects logistic longitudinal models for LGD and p53 (*i.e.* not SOX2) with the time-varying Cox model, in the framework of the multivariate joint model (referred to as ‘joint model A’). Second, to compare the estimates of the multivariate joint model of the main analysis to a more conventional analysis, a static Cox proportional hazards model was estimated (referred to as ‘Cox model’). Because in this model only baseline values were used, missing values of p53 and SOX2 were estimated by multiple imputation. For this model, the AUC was estimated at the same time points as the multivariate joint model of the main analysis, to be able to compare the performance of the static and the dynamic model.

If the model was fitted with only LGD and p53 (joint model A, S1 Table) as biomarkers, aberrant expression of p53 was estimated to be associated with a higher neoplastic progression risk than in the main analysis (main analysis HR value 1.26, p<0.01, sensitivity analysis HR value 1.55, p<0.01). Also, there was a statistically significant and slightly stronger association between having LGD and an increased risk of neoplastic progression, than in the main analysis (main analysis HR LGD accumulated effect 1.02, p=0.12, sensitivity analysis HR LGD accumulated effect 1.03, p<0.01).

These are all results of the joint models, in which estimations are based on longitudinal evolutions of histological diagnosis and immunohistochemistry. If only results of baseline are included (Cox model), the presence of LGD, aberrant expression of p53, and SOX2 are associated with an increased risk of neoplastic progression (HR LGD 3.57, p<0.01, HR p53 6.63, p<0.01; HR SOX2 2.20, p=0.04), as well as the presence of esophagitis (HR 3.38, p<0.01). The estimates of the AUC at different time points are all lower than those of the main analysis (0.72-0.78, Table 4). Consequently, the predictive performance of this static model is lower than the dynamic model. If in the same static model only baseline LGD was included and not p53 and SOX2, there was a statistically significant association with an increased risk of neoplastic progression (HR 3.40, p<0.01).
