# Supplementary material for: A personalized and dynamic risk estimation model: The new paradigm in Barrett’s esophagus surveillance
Source: PLoS One. 2022 Apr 27;17(4):e0267503. doi: 10.1371/journal.pone.0267503 (PMC9045660; doi:10.1371/journal.pone.0267503)
Supplement: S1 Fig — EAC = esophageal adenocarcinoma. LGD = low-grade dysplasia. HGD = high grade dysplasia. (DOCX) [file pone.0267503.s002.docx]

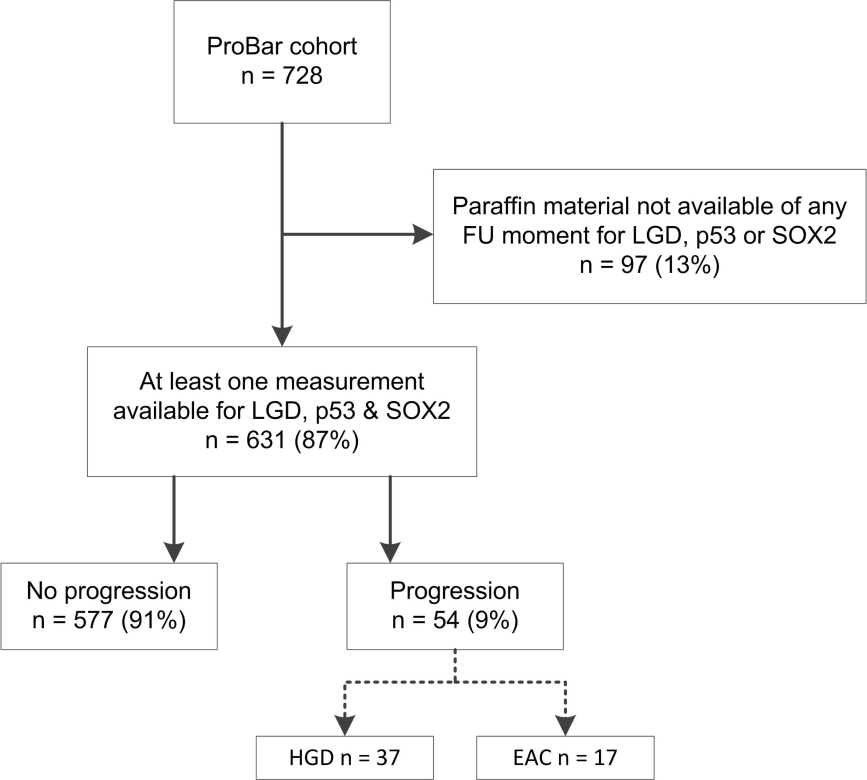


**S1 Fig.** **Flow chart of patients included.**

EAC = esophageal adenocarcinoma. FU = follow-up. LGD = low-grade dysplasia. HGD = high grade dysplasia.
