# Supplementary material for: A personalized and dynamic risk estimation model: The new paradigm in Barrett’s esophagus surveillance
Source: PLoS One. 2022 Apr 27;17(4):e0267503. doi: 10.1371/journal.pone.0267503 (PMC9045660; doi:10.1371/journal.pone.0267503)
Supplement: S1 Table — For every biomarker normal expression is the reference category. HR >1 is associated with an increased risk; HRs of joint model represent the HGD/EAC risk if the risk of aberrant expression in the longitudinal course of a biomarker changes with 10%, HRs of the Cox model represent the HGD/EAC risk if aberrant expression is present at baseline. BE = Barrett’s esophagus. HGD = high-grade dysplasia. HR = hazard ratio. LGD = low-grade dysplasia. EAC = esophageal adenocarcinoma. (DOCX) [file pone.0267503.s003.docx]

|  |  | Joint model | Joint model A  (LGD, p53) | Cox model |
| --- | --- | --- | --- | --- |
|  |  | HR (95% CI) | HR (95% CI) | HR (95% CI) |
| Age |  | 1.00 (0.98; 1.05) | 1.02 (0.99; 1.05) | 1.19 (0.86; 1.63) |
| Gender (female) |  | 1.00 (0.90; 1.02) | 1.01 (0.95; 1.04) | 0.77 (0.37; 1.58) |
| Length of BE (≥3 cm) |  | 1.02 (0.94; 1.03) | 1.00 (0.92; 1.02) | 1.04 (0.54; 2.03) |
| Esophagitis (present) |  | 1.03 (0.75; 1.02) | 0.99 (0.87; 1.03) | 3.38 (1.67; 6.85) |
| LGD | Value | 1.02 (0.47; 1.50) | 1.00 (0.63; 1.43) | 3.57 (2.01; 6.34) |
| Accumulated effect | | 1.02 (1.00; 1.06) | 1.03 (1.02; 1.05) | n.a. |
| P53 (aberrant expression) | Value | 1.26 (1.13; 1.80) | 1.55 (1.33; 2.22) | 6.63 (3.55; 12.4) |
| Accumulated effect | | 1.00 (1.00; 1.00) | 1.00 (1.00; 1.01) | n.a. |
| SOX2 (aberrant expression) | Value | 1.43 (1.26; 3.23) | n.a. | 2.20 (1.12; 4.34) |
| Accumulated effect | | 1.02 (1.00; 1.05) | n.a. | n.a. |

**S1 Table.** **Risk of neoplastic progression. For every biomarker normal expression is the reference category. HR >1 is associated with an increased risk; HRs of joint model represent the HGD/EAC risk if the risk of aberrant expression in the longitudinal course of a biomarker changes with 10%, HRs of the Cox model represent the HGD/EAC risk if aberrant expression is present at baseline.**

BE = Barrett’s esophagus. EAC = esophageal adenocarcinoma. HGD = high-grade dysplasia. HR = hazard ratio. LGD = low-grade dysplasia. N.a. = not applicable.
