# Supplementary material for: A personalized and dynamic risk estimation model: The new paradigm in Barrett’s esophagus surveillance
Source: PLoS One. 2022 Apr 27;17(4):e0267503. doi: 10.1371/journal.pone.0267503 (PMC9045660; doi:10.1371/journal.pone.0267503)
Supplement: S2 Table — (DOCX) [file pone.0267503.s004.docx]

|  | **Subsequent endoscopy** | |
| --- | --- | --- |
| **Previous endoscopy** | **normal** | **aberrant** |
| **normal** | 578 | 84 |
| **aberrant** | 61 | 135 |

**S2 Table. Absolute measurements of the variability between normal and aberrant expression for p53, not related to individual patients.**
